# Adjuvant Biophysical Therapies in Osteosarcoma

**DOI:** 10.3390/cancers11030348

**Published:** 2019-03-12

**Authors:** Valeria Carina, Viviana Costa, Maria Sartori, Daniele Bellavia, Angela De Luca, Lavinia Raimondi, Milena Fini, Gianluca Giavaresi

**Affiliations:** IRCCS—Istituto Ortopedico Rizzoli, via Di Barbiano 1/10, 40136 Bologna, Italy; viviana.costa@ior.it (V.C.); maria.sartori@ior.it (M.S.); daniele.bellavia@ior.it (D.B.); angela.deluca@ior.it (A.D.L.); lavinia.raimondi@ior.it (L.R.); milena.fini@ior.it (M.F.); gianluca.giavaresi@ior.it (G.G.)

**Keywords:** osteosarcoma, biophysical stimuli, adjuvant therapies

## Abstract

Osteosarcoma (OS) is a primary bone sarcoma, manifesting as osteogenesis by malignant cells. Nowadays, patients’ quality of life has been improved, however continuing high rates of limb amputation, pulmonary metastasis and drug toxicity, remain unresolved issues. Thus, effective osteosarcoma therapies are still required. Recently, the potentialities of biophysical treatments in osteosarcoma have been evaluated and seem to offer a promising future, thanks in this field as they are less invasive. Several approaches have been investigated such as hyperthermia (HT), high intensity focused ultrasound (HIFU), low intensity pulsed ultrasound (LIPUS) and sono- and photodynamic therapies (SDT, PDT). This review aims to summarize in vitro and in vivo studies and clinical trials employing biophysical stimuli in osteosarcoma treatment. The findings underscore how the technological development of biophysical therapies might represent an adjuvant role and, in some cases, alternative role to the surgery, radio and chemotherapy treatment of OS. Among them, the most promising are HIFU and HT, which are already employed in OS patient treatment, while LIPUS/SDT and PDT seem to be particularly interesting for their low toxicity.

## 1. Introduction

Osteosarcoma (OS) is the most frequent malignant bone-tumor, accounting for 20% of primary bone cancers, with a manifestation peak occurring during the second and third decades of life. Current standard treatment consists of surgery associated to chemotherapy, which leads to long-term disease-free survival in approximately 60% of patients with localized extremity lesion [1,2,3] and 20–30% of patients with axial primaries or metastases [1,3]. However, the efficacy of these therapeutic strategies is limited and some of them and can cause severe complications and adverse effects [4,5,6].

Traditionally, the gold standard for primary bone malignancies localized in extremity has been amputation. Over the past decades, the therapy has shifted toward limb salvage with intact local function in order to improve the life quality of patients, leading recently to the use of this protocol as a standard procedure for primary bone malignancies. Limb salvaging procedures may include resection of the local bone lesion, as well as bone reconstruction after surgery. Bone reconstruction often involves the implantation of large-segment prostheses [7,8,9,10], which are widely used with good functional results. However, this procedure has some important disadvantages in terms of clinical and economic aspects such as prosthetic loosening and periprosthetic infections or the requirement of a custom-made prosthesis design that has been recently overcome, thanks to 3D-bioprinting techniques [11,12]. Regarding the use of bone allograft for reconstruction procedures, even though its implantation is technically easier, it is strongly associated with risks of disease transmission (i.e., hepatitis, HIV, etc.), immune rejection, nonunion, and bone resorption [13,14].

**Figure 1:**
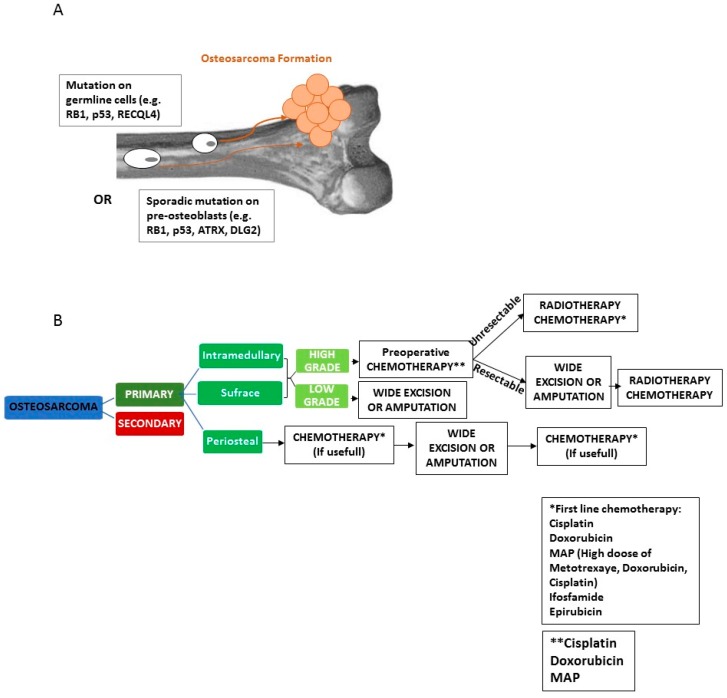
OS genesis (**A**). Genetic alterations in germline or sporadic mutation in osteoblasts interfere with the osteogenic process, resulting in an alterate balance between proliferation and differentiation, that cause an uncontrolled cell proliferation. Standard OS treatment Flow Chart (**B**) according to the NCCN Clinical Practice Guidelines in Oncology Version I.2018.

In parallel with surgical problems, there are also those related to chemotherapy ones (Figure 1). Although survival rate has increased up to 60–70% within the last 20 years, the problem of non-response to chemotherapy still remains, as well as that of toxic side effects [4,6]. For these reasons, alternative strategies able to improve the efficacy of chemotherapy and the quality of life of patients, particularly for those cases where it is impossible to perform surgery, are mandatory. In recent years, the potentialities of biophysical treatments in OS have been taken into account and several approaches have been investigated such as hyperthermia, high intensity focused ultrasound, low intensity pulsed ultrasound and sono- and photodynamic therapies. The aim of the present review was to summarize the state of the art of these different biophysical approaches to OS therapy during the last 10 years.

## 2. Search Strategy

The following literature search was carried out in the MEDLINE database (PubMed research engine) to identify studies reporting the use of biophysical therapies to treat OS, including original articles in English from January 2007 to December 2018. The keywords used were osteosarcoma, hyperthermia, high intensity focused ultrasound, low intensity pulsed ultrasound, sono- and photodynamic therapies. Two reviewers manually assessed the title and abstract of each collected reference. A total of one hundred sixty-nine articles were retrieved. However, some were not considered because they were related to: (a) canine diseases (*n* = 12); (b) the development of nanoparticles used in association with biophysical therapies or the use of ultrasounds for inducing osteogenic differentiation (*n* = 27); or (c) others that were completely non-related (*n* = 30). The resulting references were selected for supplementary analysis based on the title and abstract, resulting in 100 articles considered eligible for the review. An additional 63 articles (*n* = 35 published before 2007 and *n* = 28 from 2007 to 2018) were included in the final review to complete the introduction and conclusion section or to add information on some technical aspects.

## 3. Physical Principle of Biophysical Stimuli

### 3.1. Hyperthermia (HT)

**Figure 2:**
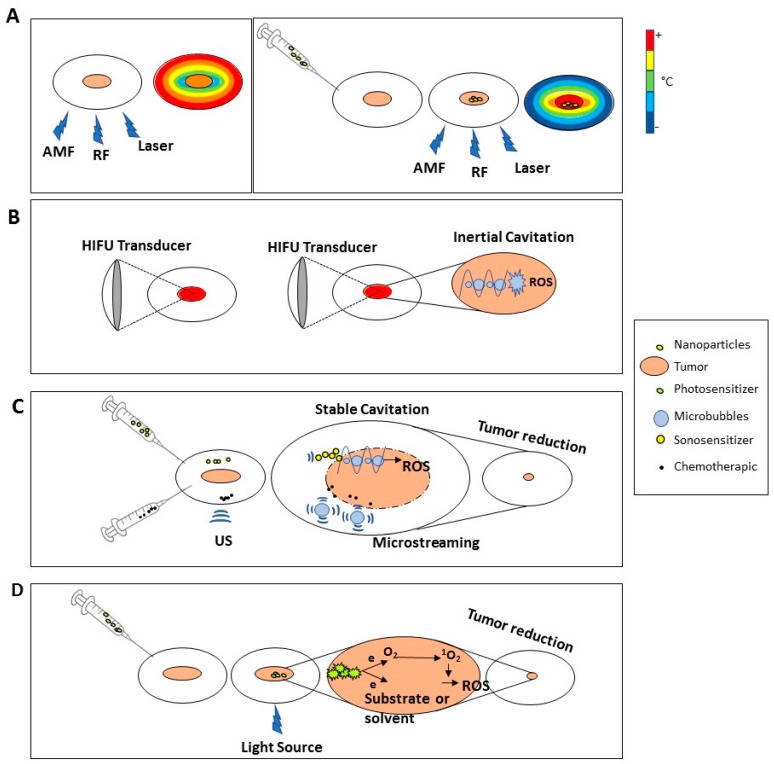
Physical principles of adjuvant biophysical stimuli. HT treatment (**A**): The use of different type of HT source alone (alternating magnetic field (AMF) or radiofrequency (RF) or laser) causes a temperature increase reaching the maximum on the body surface that decreases while moving away from the source (Left Panel). The combined use with nanoparticles is able to concentrate the heat into target cells without heating the surrounding tissues (Right Panel). HIFU treatment (**B**): Thermal effect: the mechanical energy is converted into heat reaching the maximum temperature on tumor by focusing the US in a site-specific manner (Left Panel). Mechanical effect: focused ultrasound causes inertial cavitation, bubble explosion and ROS formation (Right Panel). LIPUS and SDT Treatment (**C**): LIPUS brings microstreaming improving membrane permeability and drugs uptake, moreover, in combination with sonosensitizer, triggers stable cavitation causing ROS generation. PDT treatment (**D**): The light beam excites photosensitizer electrons that, returning to the basal level, transmit the energy to nearby molecules inducing two type of reaction: (a) generation of reactive oxygen species (ROS) into the medium; (b) activation of singlet oxygen (^1^O_2_) promoting also ROS production.

HT is an artificial increase of temperature in target cells within a range of 39–43 °C. At tissue level, this increase modifies the vascular permeability, enhances blood flow and could lead to oxygenation of the tumor, making the cancer cells more susceptible to other treatment modalities (thermal cell sensitization). Conventional HT generates a temperature gradient with a maximum on the body’s surface that decreases while moving away from the source; thus, the majority of energy is dissipated in the healthy tissues situated along the path of external radiation without any discrimination between the targeted tissue and the surrounding normal tissues, leading to serious side effects [15]. To avoid this drawback, continuing efforts to develop more effective hyperthermia methods have led to the application of nanoparticles as hyperthermia agents, which, when evenly spread in the tumor may distribute heat homogeneously. The temperature increase can be raised applying different sources (Figure 2A): electromagnetic waves (microwaves, radio waves), laser or acoustic waves (ultrasound):

- Electromagnetic waves: To achieve the desired heat in target site, magnetic nanoparticles (MNPs) are first injected and subsequently exposed to an alternating magnetic field (AMF). The AMF frequency ranges from several KHz up to 10 MHz with sufficient penetration depth. The HT efficiency is affect by several parameters: frequency an amplitude of AMF, and size-dependent magnetic properties of the nanoparticles [16].
- Radio Frequency (RF): Non-ionizing radiation are employed as an adjuvant therapy to enhance the chemotherapy and radiotherapy effects. RF waves effectively penetrate into the deep sites by needle insertion directly into the tumor site, but this technique enhance the temperature in a non-specific and non-uniform manner causing hot spots within overlying healthy tissues. To avoid these disadvantages, it is useful the use of nanoparticles (gold nanoparticles) that reach the tumor site and release RF exposure. The heating rate is inversely proportional to particle size.
- Laser: localized hyperthermia is achieved by introduction of nanoparticles (such as gold nanoparticles) into the target site where the laser exposure causes a change in the medium photothermal properties and increases the local conversion of optical energy into heat, by exciting the PS electrons [17]
- Acoustic: see High Intensity Focused Ultrasound section

### 3.2. High Intensity Focused Ultrasound (HIFU)

HIFU is a form of ultrasound delivering high intensity (>3 W/cm^2^) and high frequency (1–20 MHz) [17]. HIFU acts both by thermal and non-thermal mechanisms causing cell death at tissue level through the conversion of mechanical energy into heat (up to 80–90 °C within tissues) and unstable cavitation (formation and immediate and violent collapse of gas-filled bubbles) causing cell death acting on cell membrane and organelle rotation (Figure 2B) [18]. HIFU transducers are made by piezoelectric materials that oscillate upon alternating voltage application that cause the ultrasound waves formation in the receiving medium. The transducers employ relatively high levels of power and localize acoustic energy in a small volume in medical applications. Focusing can be reached, either by using a curved (spherical section) transducer or by using a plane transducer and a curved lens.

### 3.3. Low Intensity Pulsed Ultrasound (LIPUS) and Sonodynamic Therapy (SDT)

LIPUS is a form of ultrasound delivering low intensity (<3 W/cm^2^) and low frequency (20–200 kHz). LIPUS acts exerting both a minimal thermal effect due to its low intensity and pulsed output mode, and non-thermal effects (stable cavitation) (Figure 2C). The thermal effect is due to the absorption of US by the tissue, while the non-thermal one (mechanical effect) is due to the acoustic streaming and stable cavitation [19] which is the formation of gas bubbles caused by the accumulation of dissolved gas in the medium [20]. The acoustic streaming is responsible of membrane permeability, diffusion rate and alteration of protein synthesis, cellular secretion, and sonoporation, while the cavitation improves drugs transport and cellular up-take [17]. The sound wave is produced by a sound source, often a circular ceramic disk exhibiting a piezoelectric effect, that vibrate sinusoidally thus generating a sound wave

An important characteristic is the possibility of being used in combination with sonosensitizers, agents able to increase the energy deposition in a target area by affecting the acoustic environment [21]. The advantage of this technique is in its ability to focus the ultrasound energy on targeted tissues inducing local cytotoxicity by activating sonosensitizers with minimal damage to healthy tissues [22,23]. In SDT, the sonication parameters (usually 1.0–2.0 MHz at an intensity of 0.5 to 3.0 m^2^) are selected to produce inertial cavitation in a cell culture or tumor [24]. 

### 3.4. Photodynamic Therapy (PDT)

PDT (Figure 2D) is a treatment based on the local light application after systemic photosensitizer (PS) injection [25]. The light sources are characterized by two factors: a wavelength ranging between 600–800 nm and illumination intensity (in the near infrared spectral region-NISR) that do not cause damage to tissues [26]. The penetration capability depends on wavelengths, as the effective illumination intensity is too weak for deep tissue, indeed the major challenge is to find a novel and appropriate irradiation approach, or apply its use in an intraoperative setting [26]. PDT exploits the ability of photosensitizers to release energy to nearby molecules after excitation with specific light wavelengths, transforming light energy into chemical energy. The PSs are molecules characterized by two important features: (a) they are non-toxic to normal tissue in the dark, and (b) are able to cause photodamage with an appropriate light source without temperatures rising, distinguishing PDT from photothermal therapy [27,28]. Each PS has an exciting light with optimum wavelength that is able to excite PS electrons that return to the basal level transmitting energy to nearby molecules inducing two type of reaction: (a) generation of reactive oxygen species (ROS); (b) activation of singlet oxygen (^1^O_2_) promoting also ROS production. Both mechanisms are able to cause cell apoptosis [25,29,30,31]. 

## 4. Biophysical Therapies in Oncology

### 4.1. HT

HT acts at cellular level through several mechanisms: proteins denaturation, DNA and RNA damages, reactive oxygen species (ROS) production [32], heat shock proteins (HSPs) activation [33,34,35], and intrinsic and extrinsic apoptosis pathways activation [32,36]. Currently HT is considered an adjuvant therapy in treating numerous cancers [37] when associated to radio- or chemotherapy, thanks to new technology that can provide precise control and measurement of heat delivery [33,34,35,38,39,40,41]. In fact, HT treatment can be modulated to control tissue temperature around 40–42 °C, which does not determine cell death and vascular destruction, but increases cancer cell sensitivity to radiotherapy or various chemotherapeutic drugs, and tumor blood perfusion, improving drug delivery to tumor cells [42]. HT may alter the membranes of the tumor cells enabling the drugs to penetrate them more easily. Moreover, HT may also promote the ability of the drugs to induce apoptosis in the tumor cells, thanks to molecular pathways modulation [43,44]. Indeed, a randomized phase III trial showed that the combination of regional HT with neoadjuvant chemotherapy for soft tissue sarcomas had better local progression-free survival than chemotherapy alone [45].

HT can be also combined with radiotherapy representing a possible tool for local control of inoperable tumors or an adjuvant therapy in the context of surgery. Heat radiosensitization is due to the pleiotropic effect as a damaging agent on multiple cell components by altering protein structures and/or influencing the DNA damage response [46,47,48]. Radiofrequency and ultrasound HT ablations were reported as further HT modalities, but their efficacy depends on the size and depth of the tumor, and disadvantages include the ability to target the tumor and control the exposure [49].

A promising way to overcome these disadvantages might be the combined use of HT and nanoparticles able to localize specifically inside a tumor, producing an increase in temperature higher than the surrounding tissues, leading to cell death [50,51]. The heat dissipation can be exploited from MNPs under an alternating current magnetic field in specific tumor sites, resulting in a therapeutic outcome and driving malignant cells to destruction, in particular for cancer cells that are generally more susceptible in regional temperature variations than normal cells [52].

Several studies were performed on gold nanoparticles, such as nanospheres and gold nanorods for tumor treatment, providing remarkable opportunities in the detection and therapy due to their inherently low toxicity [53] and strongly enhanced optical properties associated with localized surface plasmon resonance (LSPR) [54,55]. These characteristics allow the local temperature to increase to more than 50 °C, causing the so-called thermal ablation, which corresponds to severe cell damage resulting in coagulative necrosis and membrane lysis [56].

Moreover, an important aspect of the combination of HT to MNPs is to allow the release of the selected drug loaded on MNPs in cancer tissue when they are irradiated with an appropriate laser beam [57,58,59], thus increasing drug delivery efficiency and favoring intracellular drug incorporation, thanks also to the temperature-induced increase of cell membrane permeability [60].

### 4.2. HIFU

Recent studies on acoustic technology have shown how ultrasounds can be an important resource not only for diagnosis, but for oncological therapy as well [17,61]. In particular, HIFU is usually employed in cancer therapy for tumor ablation [62,63] and pain reduction [64]. 

It has been used for various kinds of malignancies, including prostate, liver, breast, kidney, pancreas, bone metastasis, glioblastoma and soft tissue sarcoma [65,66,67,68,69,70,71,72,73]. In recent years, the HIFU technique has been used more because its potential has been improved by the combined use of imaging equipment. Currently, both B-mode ultrasonography (US) and magnetic resonance imaging (MRI) have been incorporated into HIFU devices developing US-imaging-guided HIFU (USgHIFU) and MRI-guided HIFU (MrgFUS) and facilitating the ablation of a three-dimensional target [74,75]. Recently, the MRgFUS has been approved by the FDA to be used in bone metastasis to treat pain management [76].

### 4.3. LIPUS and SDT 

It is well known that mechanical stimuli, such as ultrasounds, in the bone microenvironment are important for bone homeostasis and growth, playing key roles in the development of many tissues such as bone, cartilage or lung [77,78,79]. Ultrasounds at lower intensity can also play a role in developing a mild temperature, which enhances blood flow and increase vascular permeability useful in both bone regeneration and tumor treatment. In this regard, the increase in temperature and oxygenation promotes the effectiveness of chemotherapy [45]. In oncology, LIPUS treatment has been studied for several therapeutic uses [80]: (a) Sonodynamic Therapy (SDT): combination of LIPUS and sonosensitizers to affect cancer cells [81,82,83]; (b) ultrasound-mediated chemotherapy: combination of LIPUS and chemotherapeutic molecules that increase their activity in cancer therapy [84,85,86]; (c) sonoporation: technique used both to affect cells directly and for gene delivery or transfection [87,88] and (d) molecular effects on cancer cells [81,82,83,89,90].

In recent years, the use of SDT in tumor treatment has increased, demonstrating its ability to mediate apoptosis in numerous experimental systems in vitro or in vivo. The capability of SDT to induce cell death by apoptosis was proven in different human tumor cell lines, such as liver, oral, leukemia, lung and colon cancer cell lines [37].

### 4.4. PDT

PDT induces tumor destruction by cellular effects, vascular effects or both. Cellular lethal effect may be caused by an imbalance in the mitochondria, lysosomes, plasma, hydrolytic enzymes, certain cytokines and calcium, or even by DNA damage [91]. As a result, the tumor cells are eliminated through apoptotic pathway or necrosis.

Despite its approval almost twenty years ago by the FDA, PDT is nowadays only used to treat a limited number of cancer types (skin, bladder) and non-oncological diseases (psoriasis, actinic keratosis) [25]. PDT was commonly used for the treatment of solid tumors at superficial anatomical locations (head and neck cancers, skin cancer, and malignant melanomas) due to the limited light penetration capacity [25,92]. Its application in other tumors at more inaccessible sites is still an aim of clinical investigations.

Another important advantage of PDT is its ability to bypass multidrug-resistance (MDR) in various deep tumor models, which is the main limitation of prognosis improvement in cancer patients [93,94], by means of: (a) inhibiting anti-apoptotic proteins [95]; (b) preventing a drug-efflux effect, impairing ATP-binding transporters [96]; (c) altering the microenvironment of tumor cells, including microvascular injury and inflammatory factor secretion [97,98]; (d) enhancing the permeability of tumor vessels and promoting drug delivery [98,99] and (e) promoting immune system response [100]. Finally, in addition to elicit direct cytotoxicity, PDT provokes a variety of additional beneficial anti-tumor effects such as an acute inflammatory response, anti-vascular effects and an activation of the immune system [25].

An important disadvantage of PDT therapy concerns the PSs distribution in the body. Despite nanotechnology and other targeting techniques, PSs still tend to concentrate in the liver, kidney and other tissues [101,102]. The non-specific concentration of PSs leads to irradiated injury of normal tissues as well as liver and kidney damage. Since the existing PSs are not satisfactory for further PDT development, there is a need for another generation of PSs [31].

## 5. Application of Biophysical Therapies in Osteosarcoma

### 5.1. HT

HT induced by microwave energies is already used in orthopedics to treat bone tumors during surgery [103,104]. The cellular responses to HT are different and depend mainly on temperature and protocols employed (Table 1). Therefore, it is mandatory to investigate and know the correct temperature to be used in different tumors, because just a degree of difference might change strongly the effect of treatment [105,106]. One of the mechanisms by which HT causes cell death involves the heat shock proteins (HSPs) pathway and the different expression level of HSPs in different tumor cells seems to be one of the major reasons for the variances in thermo-sensitivity [33,34,35]. Some in vitro studies have shown that HT treatment inhibited cell proliferation in OS cell lines via HSP70 upregulation [36], reduced tumor cell motility and autocrine motility factor (AMF) expression via HSP27 [107], and induced cell apoptosis via ROS, ER stress, mitochondria, and caspase pathways [32]. In a more recent work, Moise et al. showed in a preliminary study that HT treatment at 42 °C, apart from induce cell death, is able to trigger differentiation commitment towards a mature phenotype in the surviving cells, showing another important mechanism through which HT could affect tumors [108]. another recent study by Han et al. showed that microwave-induced HT may be an alternative treatment for distal tibia OS, without any apparent increase in death, local recurrence, or complications [109]. 

#### 5.1.1. HT and Chemotherapy

Concerning the antitumor action of thermo-chemotherapy, the ability of the drugs to induce apoptosis in tumor cells is mainly attributable to the molecular pathway’s modulation induced by heat (Table 1). Although it is evident that the effect of HT depends on and varies with temperature (41–45 °C), it acts differently according to administered drugs and treated cell types.

Debes et al. demonstrated that HT at 43 °C, but not at 42 °C, influenced differently OS cell viability, depending on OS aggressiveness, affecting strongly MG-63 and KHOS cell line and only weakly U-2 OS and Saos2 cell line [105]. Moreover they showed also that HT was able to enhance the cytotoxic effect of cisplatin, affecting cell viability at both temperatures, with a stronger effect at 43 °C [105]. Other studies showed that HT conditions sensitize OS cell line to paclitaxel and cisplatin or etoposide combinations by upregulating Fas expression [110,111]. The combined use of HT with b-lapachone (b-lap) or melphalan in OS resulted in the enhancement of antitumor activity upregulation of NAD(P)H: quinone oxidoreductase (NQO1) [112] or by caspase-3 activation [113].

Recently, a clinical randomized phase III trial evaluating local progression-free survival of patients with high-risk soft tissue sarcoma (including extraskeletal osteosarcoma) treated with chemotherapy (neoadjuvant etoposide, ifosfamide, and doxorubicin) with or without regional HT (NCT00003052: Combination Chemotherapy With or Without Hyperthermia Therapy in Treating Patients With Soft Tissue Sarcoma) showed that adding regional HT improved survival and local progression-free survival [45,114]. However, HT enhanced leukopenia and determined moderate adverse events such as pain, bolus pressure, and skin burn [45].

#### 5.1.2. HT and Radiotherapy

The HT capability to act as a radiosensitizer in combination with radiotherapy make it useful in inoperable OS [47,48]. Tancredi et al. showed the efficacy of HT as adjuvant treatment of surgery in a case report of a patient affected by an irradiation-induced recurrent OS [115] (Table 1). Indeed, the post-irradiation OS seems to be an important field of application, because it is not possible (1) to administer the typical chemotherapy used to treat primitive sarcomas to these patients when their general conditions do not allow it, and (2) to expose them to radiation therapy due to its side effects on a site that had already been irradiated [116,117]. 

#### 5.1.3. HT and Nano- and Magnetic Nanoparticles

In recent years, a variety of nanostructures have been exploited in the areas of OS imaging, diagnostics and treatment [118], such as magnetic nano-particles, implantable thermoseeds and gold nanoparticles (Table 1). Shido et al. showed in an in vivo model of OS that magnetite cationic liposomes (MCLs) associated to a magnetic field were able to reduce both local tumors and lung metastasis [119]. Implantable thermoseeds such as N_glass–glass ceramic [120], ferrimagnetic glass–ceramics biomaterial [121]; ferrite MNPs [122,123] or hydroxyapatite-coated iron oxide (IO-HAp) [124], all activated by an external magnetic field, permit to focus the heat into the target OS. In vitro studies showed that combined use of HT, gold nanoparticles and doxorubicin decreased survival rate of Saos2 cell line in comparison with single treatment [54]. Finally, recent studies showed the effect of the gold nanorods (folate-targeted nanorods: Au NRs@INU-LAPEG-FA/Nut and Au NRs@PHEA-EDA-FA/Nut or polyacrylic acid-coated nanorods: GNRs@PAA), acting as imaging contrast agents, effective drug delivery systems and hyperthermic agents, display a remarkable anticancer activity on both OS bidimensional and tridimensional cell cultures [125,126].

### 5.2. HIFU

The use of HIFU as an alternative treatment to surgery in OS has been debated and not thoroughly accepted [127,128,129] (Table 2). Li et al. reported for the first time in 2009 the use of HIFU as an alternative treatment in seven OS patients (perspective study) rejecting amputation or where the surgery may cause serious wounds, complications, and disabilities because of tumor location [129,130]. They found no severe complications to HIFU treatment and disappearing of preexisting severe pain in all patients. A complete response to HIFU treatment was achieved in three patients and a partial one in other three patients; the last patients developed a pulmonary metastasis 5 months after HIFU treatment. The median survival time was 68 months and the five-year survival rate was 71.4% [130]. In 2010, again Li et al. showed the advantages of HIFU treatment in a further group of 12 patients with OS [129]; patients underwent neoadjuvant chemotherapy before HIFU (4–6 weeks), followed by 2 to 4 weeks of adjuvant chemotherapy, 10 to 20 days after HIFU using high-dose methotrexate/vincristine and doxorubicin and cisplatin. In treating all patients with HIFU until complete tumor ablation, they demonstrated a significant pain reduction and improvement in alkaline phosphatase and lactate dehydrogenase markers, with an overall survival rate of 100.0%, 84.6%, 69.2% and 38.5% at 1-, 2-, 3- and 5-year, respectively [129]. They suggest that a possible related mechanism for pain relief may be the thermal periosteal denervation or the thermal ablation of the tumor tissue mass itself that could reduce the pressure on adjacent healthy tissues or a combination of these mechanisms [129]. They concluded that HIFU seems to be a safe and noninvasive technique, appearing to be successful in the treatment of OS, which cannot be surgically removed. The chemotherapy administration before and after HIFU treatment not only inhibited growth of subclinical metastatic foci, preventing recurrence, but also minimized tumor size, suggesting a synergistic effect with chemotherapy [129]. In response to the Li study, Bielack et al. [127], highlighted various concerns regarding the combined use of HIFU with chemotherapy as an alternative to surgery, which is considered the gold standard, and above all, they pointed out that newly diagnosed patients with OS were not treated with surgery in favor of a purely experimental approach [65,129,130]. In addition, Bielack et al. stated that no sufficient information about the local control rate of HIFU in primary malignant bone tumors was reported [127]. 

Other studies in favour of the use of HIFU for unresectable OS are Chen et al. Orgera et al. and Yu et al. [65,131,132]. Chen et al., carried out a prospective study from 1997 to 2004 on 80 patients with bone tumors of which 63 were affected by OS and refused to undergo surgery or were not candidates for surgery [65]. Patients underwent neoadjuvant chemotherapy (three to five cycles) and adjuvant chemotherapy (four to six cycles) before and after HIFU ablation, respectively, with cisplatin, doxorubicin, methotrexate and ifosfamide. They reported overall survival rates for all patients at 1, 2, 3, 4 and 5 years that were 89.8%, 72.3%, 60.5%, 50.5% and 50.5%, respectively [65]. Orgera et al. treated with HIFU 22 patients with different solid tumors who were deemed not to be candidates for surgery because of comorbidities. One of 22 patients was affected by OS and HIFU treatment determined a pain reduction and tumor response [131]. Yu et al., performed a retrospective analysis of 27 OS patients who had local unresectable recurrence or refused to undergo surgery or who were not candidates for surgery, which were treated with HIFU from 2006 to 2010 [132]. They concluded that HIFU might be considered a safe and noninvasive treatment for local unresectable recurrence of OS, with good local control and without severe complications, achieving an overall survival rate of 59.2%, 40.7% and 33.1% at 1-, 2-, 3-year, respectively [132].

According to these few non randomized trials, the advantage of HIFU treatment are numerous: (a) noninvasive; (b) a real-time treatment is possible by monitoring the beam by US or MRI; (c) an uniform distribution of therapeutic dose, employing the destroying treatment only on the target area [129]. On the contrary, the main complications of HIFU therapy in malignant bone tumors included skin burns in the therapy area and local nerve injury. Moreover, other potential complications are the fracture of the tumor-affected bone, the functional loss of nearby joints and the hemorrhagic infection of the tumor. Additionally, HIFU was considered inappropriate in same conditions: (a) pathological fractures; (b) tumors located in the spine or skull; (c) when the distance between tumor and skin is < 0.5 cm; (d) when tumor crosses a joint; or (e) tumor crosses/surrounds a nerve or blood vessel [65,129,130]. 

Currently, there are three ongoing nonrandomized phase 1 studies evaluating HIFU safety alone or in combination with chemotherapeutic agents:

(1) NCT02076906—MRg-HIFU on pediatric solid tumors, whose purpose is to determine if MRgFUS ablative therapy is safe and feasible for children with refractory or relapsed solid tumors;
(2) NCT02557854—HIFU hyperthermia with liposomal doxorubicin (DOXIL) for relapsed or refractory pediatric and young adult solid tumors, which aims at evaluating whether Doxil given prior to MR-HIFU hyperthermia (50 mg Doxil i.v. followed by MR-HIFU 42 °C for 30 min every four weeks) is safe for the treatment of pediatric and young adult patients with recurrent and refractory solid tumors;
(3) NCT02536183 A phase I study of lyso-thermosensitive liposomal doxorubicin and MR-HIFU for pediatric refractory solid tumors, which evaluates the maximum tolerated dose and recommended phase 2 dose of lyso-thermosensitive liposomal doxorubicin (LTLD) administered in combination with MR-HIFU in children with relapsed/refractory solid tumors. LTLD is administered i.v. in combination with MR-HIFU ablation on day 1 of every 21-day cycle, receiving up to six cycles.

### 5.3. LIPUS and SDT

Preclinical studies on LIPUS treatment in OS concern their effect on molecular pathways and, above all, their action in SDT. Matsuo et al. in an in vitro study showed that LIPUS treatment alone was able to directly cause apoptosis mitochondrial pathway dependent and necrosis in OS cells [133]. Furthermore in vitro and in vivo studies demonstrated that LIPUS treatment alone showed a cytotoxic effect on OS cell line via ROS and Ca^2+^ and that LIPUS stimulation was able to inhibit tumor growth in OS xenograft model (Table 3). In both cases, the combined use of sonosensitizer Hematoporphyrin monomethyl ether (HMME) enhanced these effects [82,83]. Indeed, the combined use of LIPUS and SDT is the most investigated and promising application in OS treatment. In vitro and in vivo studies for OS treatment showed that two sonosensitizer are able to inhibit tumor growth: HMME [82,83,134] and 5-Aminolevulinic acid (ALA) [5,135].

### 5.4. PDT

Several preclinical in vitro and in vivo studies in OS showed that PDT is able to induce apoptosis (activation of mitochondrial and/or caspase pathways, and/or ROS increase), autophagy (activation of ROS-Jnk signaling pathway) and/or to arrest cells at the G2/M phase, depending on PS employed (Table 4) [136,137,138,139,140,141,142,143,144,145,146,147,148]. In addition, PDT can be employed in combination with other biophysical stimuli: low-level light therapy (LLLT) combined with N-aspartyl chlorin e6-PDT (NPe6-PDT) to enhance the cytotoxicity in MG-63 cells by increasing ROS and ATP [149]. The association of HT with PDT may increase its effectiveness in cells that do not respond to PDT alone [150]. 

Several in vivo studies have shown the effects of PDT in association with different PS on OS. Burch et al. tested in a pilot study, the use of PDT in canine osseous tumor showing the capability of PDT to induce necrosis in a large tumor tissue. In particular, they showed the possibility to place in the center of the tumor the fiber optic without compromising the native tissue, demonstrating that the skeleton could be an ideal place for PDT [151]. Acridine Orange-PDT (AO-PDT) was able to inhibit both cell invasion and pulmonary metastases in mouse OS [152]. The PDT capability to affect OS tumor size by apoptosis was demonstrated also by Sun’s studies with hiporfin-PDT in OS [144], and benzochloroporphyrin derivative 18-PDT (BCPD18-PDT) on Ewing sarcoma [145] and by Zeng’s studies on hematoporphyrin monomethyl ether-PDT (HMME-PDT) combination in mouse models [148]. Furthermore, the association of PDT with BCPD-17 suppresses local recurrence after tumor resection [136]. The effects of PDT combined with different photosensitizer are confirmed by Yu et al. [153,154] studies, showing that ZnPc/BSA-PDT (zinc phthalocyanine BSA conjugated) and PPZ-PDT (PEG-PMAN/ZnPC) were able to reduce tumor size when used intraoperatively; moreover they were able to reduce cell invasiveness in vitro. suggesting their potential effect on tumor recurrence. Meier et al. demonstrated the tumor suppressive effects in two clinically relevant intra tibial mouse models of OS of 5,10,15,20-tetrakis (*meta*-hydroxyphenyl)chlorine-PDT (mTHPC-PDT), using both mTHPC (Foscan) and a liposomal mTHPC formulation (Foslip) [155]. The authors also confirmed the potential of PDT to inhibit lung metastatic growth in animals with an intact immune system, validating the pioneering Korbelik’s studies on the importance of an intact immune system for the efficacy of PDT. De Miguel et al. showed that 5,15-bis(3-hydroxyphenyl) porphyrin-PDT was able to reduce cranial and vertebral osteosarcoma in a mouse model, suggesting that PDT is a potential antitumoral treatment for surgically inoperable osteosarcoma [156]. 

An important advantage of PDT that might be fundamental in OS therapy is its ability to bypass MDR, which is one of the main unresolved problems in OS. Kusuzaki et al. showed that AO-PDT has a strong cytocidal effect, not only on chemosensitive mouse OS cells, but also on MDR mouse OS cells [157]. This effects on MDR were confirmed by Martella et al. [158] in vitro study demonstrating that the combination of PTX-Ce6@Ker (paclitaxel loaded in keratine nanoparticles functionalized with the photosensitizer Chlorin-e6) and PDT was highly effective on MDR Saos2 cells. A recent interesting application of PDT is its combination with doxorubicin engineered osteogenic scaffold that would allow to act simultaneously destroying the tumor by increasing the temperature and the release of doxorubicin (after laser stimulation), and promoting bone regeneration thanks to the osteoinductive characteristics of the scaffold [159,160]. A clinical trial on 10 patients affected by primary musculoskeletal sarcomas (two with OS), treated after limb salvage surgery with PDT using AO-PDT, showed that: (a) the limb function recovered to the level before surgery, except for one patient; and (b) no patients showed local or systemic complications, suggesting that AO-PDT may be a promising new limb salvage modality for preservation of excellent limb function in patients with musculoskeletal sarcoma [161]. By considering that PDT has a relatively low systemic toxicity, repetitive application is possible making PDT an interesting novel option for the treatment of OS, especially in combination with current standards of care including neoadjuvant chemotherapy. 

## 6. Conclusions

The current review highlighted how, during the last decade, the technological development of biophysical therapies might represent an adjuvant role and, in some cases, an alternative role to the surgery, radio and chemotherapy treatment of OS. Among reported biophysical therapies, the most promising as adjuvant therapies are HIFUs and HT, which are already employed in OS patient treatment, while LIPUS/SDT and PDT seem to be particularly interesting for their low toxicity. However, all studies for their use in OS are still at the pre-clinical level. An important advantage of SDT and PDT is their use in target therapy that limits non-specific toxicities because of directing against cancer-specific molecules and signaling pathways, but until now, sono- and photo-sensitizers research, as well as the lack of technology, limited their clinical application, although recent studies in the field of PDT have had a strong increase, suggesting its use particularly in intraoperative phase.

On the contrary, the use of biophysical therapies as alternatives to surgery in OS has been widely debated. The prognosis of OS patients have improved markedly following the introduction of effective chemotherapy, but the prognosis for unresectable primary OS arising in the axial skeleton or with distant metastases still remains poor [162,163]. 

The prospects of adjuvant biophysical therapies in OS might be different according to the type of physical stimuli. Thanks to randomized clinical trials, which might provide evidence of HIFU and HT efficacy as alternative therapies to surgery in OS, these treatments in combination with chemotherapeutic agents could represent a treatment solution for inoperable lesions and in cases of recurrence due to MDR acquisition. Regarding SDT and PDT, these are relatively new technologies, which need further improvements to overcome their limited clinical application. They have the important advantage of being able to use them in target therapy that limits non-specific toxicities, because of directing against cancer-specific molecules and signaling pathways. After having improved these technologies, they should be investigated in clinical trials of phase 0–2, to first provide information on their safety. Although, recent studies in the field of PDT have had a strong increase, suggesting its use particularly in intraoperative phase, further extensively investigations on PS and their activation in deep tumors are mandatory in order to translate these technologies from bench to bedside and to start clinical trials.

## Figures and Tables

**Table 1:** Selected preclinical and clinical studies on HT treatment for osteosarcoma.

Study	Cell/Animal Models Patients	Treatment	Mechanisms/Results	Reference
In vitro	HOS85, MG-63 Saos2	HT 42 °C	Cell viability reduction HSP70-dependent alkaline phosphatase activity-dependent	Trieb et al., 2007 [36]
	HuO9	HT 41 °C	Cell viability reduction Hsp27-dependent, AMF-dependent	Nakajima et al., 2012 [107]
	U-2 OS	HT 43 °C	ROS; apoptosis ER stress mitochondria, caspase mediated	Hou et al., 2014 [32]
	MG-63	HT 42 °C HT 47 °C	Cell death at both temperatures. Triggering of cell differentiation commitment even at 42 °C	Moise et al., 2018 [108]
	MG-63, KHOS, U-2 OS Saos2	HT 43 °C HT 43 °C + Cisplatin HT 43 °C + Etoposide	Cytotoxicity	Debes et al., 2005 [105]
	RD- ES (primary Ewing’s sarcoma)	HT 42 °C + Melphalan	Apoptosis Caspase 3 dependent	Krause et al., 2008 [113]
	HOS	HT 42 °C + β-lapachone	Cytotoxicity NQO1-dependent	Hori et al., 2011 [112]
	OS732	HT 43 °C + Paclitaxel+ Etoposide	Apoptosis Fas-dependent	Huang et al., 2012 [110]
	OS732, MG-63	HT 43 °C + Paclitaxel+ Cisplatin	Apoptosis Fas-dependent	Huang et al., 2013 [111]
	LM8 subcutaneous in syngeneic host mouse	HT 45 °C (Alternating Magnetic Field) + MCLs	Cytotoxicity Hsp70	Shido et al., 2010 [119]
	Saos2	HT 41–43 °C (Alternating Magnetic Field) + glass–glass ceramic thermoseeds	Apoptosis	Alcaide et al., 2012 [120]
	HOS	HT 45 °C (Magnetic field) + magnesium–calcium ferrites nanoparticles	Cytotoxicity	Saldívar-Ramírez et al., 2014 [123]
	Saos2	HT 45 °C (Magnetic Field) + ferrite magnetic nanoparticles	Cytotoxicity	Makridis et al., 2016 [122]
	Saos2	HT (Magnetic Field) + ferrimagnetic glass–ceramics nanocomposites	Cytotoxicity	Gamal-Eldeen et al., 2017 [121]
	MG-63	HT 45 °C (Magnetic field) + Hydroxyapatite Coated Iron Oxide Nanoparticles	Cytotoxicity	Mondal et al., 2017 [124]
	Saos2	HT 42 °C (Microwave) + gold nanoparticles and doxorubicin	Cytotoxicity	Ghahremani et al., 2011 [54]
	U-2 OS (2D and 3D cultures)	HT (laser beam) Folate-targeted gold nanorods	Cytotoxicity	Li Volsi et al., 2017 [125]
	MG-63	HT (laser beam) PAA- coated nanorods	Cytotoxicity and apoptosis	Pan et al., 2018 [126]
Clinical	Patients Case report Irradiation-induced recurrent OS	Surgical resection followed by radiation therapy combined with HT	Results: Five months after the surgery, the clinical and instrumental control showed an effective consolidation of the chest wall and good trophism of the flap without recurrence.	Tancredi et al., 2011 [115]
	Patients Retrospective study 79 patients with distal tibia OS without metastasis	HT 52 patients were treated with microwave-induced hyperthermia, 27 patients were treated with amputation	Results: Local recurrence and survival comparable with amputation treatment. Function improvement compared with transtibial amputation. Complication: 6/52 patients hyperthermia treated experienced same complications: 2 delayed union; 1 fracture; 2 superficial infections; 1 deep infection. 3/27 patients undergoing amputation experienced complication: 2 wound dehiscence; 1 superficial infection.	Han et al., 2017 [109]
	Patients Clinical trial Randomized phase 3 340 patients with soft tumor sarcoma	HT + etoposide, ifosfamide, and doxorubicin	Results: Compared with neoadjuvant chemotherapy alone, adding regional hyperthermia improved local progression-free survival and 5-year survival rate of 62.7% vs. 51.3% and 10-year survival of 52.6% vs. 42.7%	Issels et al., 2010, 2018 [45,114]

Abbreviation; HIFU High Intensity Focused Ultrasound; US Ultrasound.

**Table 2:** Selected clinical studies on HIFU treatment for osteosarcoma.

Clinical Study	Treatment	Mechanisms/Results	Reference
Patients: 7	HIFU	Results: Complete response in three patients Partial response in three patients Pulmonary metastasis after 5 months in one patient five-year survival rate was 71.4% Severe pain disappeared Complications: No severe complications were observed	Li et al., 2009 [130]
Patients: 25 patients with malignant bone tumors; 12 with OS	HIFU + chemotherapy	Results: Tumor ablation The response rate based on MRI or PET/CT for patients with primary bone tumors was 84.6%; for patients with metastatic bone tumors, response rate was 75.0%. Pain was significantly alleviated Complications: 12 patients had first-degree burns Two patients had second-degree burns.	Li et al., 2010 [129]
Patients: Retrospective study on 80 patients with a primary bone malignancy and 60 with OS	US-HIFU + chemotherapy in 62 patients with OS, 1 with periosteal osteosarcoma, and 3 with Ewing sarcoma. US-HIFU alone in 14 patients with chondrosarcoma, giant cell bone cancer, periosteal sarcoma, or an unknown malignancy	Results: Tumor ablation in 69 patients malignant bone tumors resulted completely ablated and the remaining 11 patients showed greater than 50% tumor ablation For all patients the overall survival rates at 1, 2, 3, 4 and 5 years that were 89.8%, 72.3%, 60.5%, 50.5% and 50.5%, respectively Complications: Mild local pain and local edema after treatment; skin toxicity 17 of the 80 study patients Bone fracture was observed in six patients, ligamentous laxity occurred in three, and epiphysiolysis or secondary infection occurred in two.	Chen et al., 2010 [65]
Patients: 22 patients with solid tumors, 1 with OS	US-HIFU	Results: Tumor ablation, pain reduction Complications: No complications detected in the patient with OS	Orgera et al., 2011 [131]
Patients: Retrospective study on 27 patients with local unresectable recurrence of OS previously subjected to multi-agent chemotherapy	HIFU	Results: Tumor ablation; Pain reduction Follow up: For all patients, the 1-, 2- and 3-year local disease control rates were 59.2%, 40.7% and 33.1%, respectively Complications: Low grade fever in six patients.	Yu et al., 2015 [132]

Abbreviation; LIPUS Low Intensity Pulsed Ultrasound, HMME Hematoporphyrin Monomethyl Ether, ALA 5-Aminolevulinic acid, ROS Reactive Oxygen Species.

**Table 3:** Selected preclinical studies on LIPUS treatment for osteosarcoma.

Study	Cell/Animal Models Patients	Treatment	Mechanisms/Results	Reference
In vitro/in vivo	In vitro (MG-63 cells)	LIPUS + HMME	Apoptosis Caspase dependent	Liu et al., 2015 [134]
	In vitro (UMR-106 cells)	LIPUS alone LIPUS + HMME	Cytotoxicity ROS and Ca^2+^ dependent	Tian et al., 2010 [83]
	In vitro (LM8 cells)	LIPUS	Apoptosis and necrosis	Matsuo et al., 2017 [133]
	In vitro (UMR-106 cells)	LIPUS + 5-ALA	Apoptosis mitochondrial pathway dependent	Li et al., 2015 [135]
	In vitro (UMR-106 cells) In vivo (mouse)	LIPUS + 5-ALA	Apoptosis ROS mitochondrial pathway dependent	Li et al., 2015 [5]
	In vivo (mouse)	LIPUS alone LIPUS + HMME	Apoptosis	Tian et al., 2009 [82]

Abbreviation; LIPUS Low Intensity Pulsed Ultrasound, HMME Hematoporphyrin Monomethyl Ether, ALA 5-Aminolevulinic acid, ROS Reactive Oxygen Species.

**Table 4:** Selected preclinical and clinical studies on PDT treatment for osteosarcoma.

Study	Cell/Animal Models Patients	Treatment	Mechanisms/Results	Reference
In vitro	In vitro MOS/ADR1	AO-PDT	Cytotoxic effect on OS MDR cells	Kusuzaki et al., 2000 [157]
	In vitro HOSM-1, HOSM-2	Aminolevulinic acid hexyl ester-PDT (hALA-PDT) hALA-PDT + HT (43.5 °C)	hALA-PDT + HT treatment enhances the reduction of cell viability in cells insensitive to hALA-PDT alone	Yanase et al., 2009 [150]
	In vitro 143B	mTHPC-PDT	Apoptosis caspases- dependent in metastatic cell line	Reidy et al., 2012 [143]
	In vitro UMR106	Methylene blue-PDT	Apoptosis mitochondrial pathway induced	Guan et al., 2014 [137]
	In vitro Hu09	na-pheophorbide-PDT	Apoptosis mitochondrial and caspase pathways dependent	Nagai et al., 2014 [142]
	In vitro MG-63	NPe6-PDT + LLLT	Cytotoxicity ROS and apoptosis dependent	Tsai et al., 2015 [149]
	In vitro MG-63	ALA-PDT	Cytotoxicity	Li et al., 2016 [140]
	In vitro MG-63	Pyropheophorbide-α methyl ester-PDT	Apoptosis mitochondrial pathway induced Autophagy ROS-Jnk dependent	Huang et al., 2016 [138]
	In vitro MG-63	Aloe-emodin-PDT	Autophagy, apoptosis ROS-JNK induced	Tu et al., 2016 [146]
	In vitro MG-63	ALA-PDT	Cytotoxicity	White et al., 2016 [147]
	In vitro MG-63	TiO_2_ @xGd NBs-PDT	Cytotoxicity ROS induced	Imani et al., 2017 [139]
	In vitro MG-63, U2OS, Saos2, Saos2/^DX580^	PTX-Ce6@Ker-PDT	Increase of cell death both 2D and 3D cell model systems, and in MDR Saos2 cell line	Martella et al., 2018 [158]
In vitro and In vivo	In vitro LM-8 In vivo (mouse)	Methylene blue-PDT	Apoptosis Necrosis	Matsubara et al., 2008 [141]
	In vitro LM8 In vivo (mouse)	AO-PDT	Cell invasion and pulmonary metastases inhibition	Satonaka et al., 2011 [152]
	In vitro LM8 In vivo (mouse)	BCDP-17-PDT	Apoptosis Local recurrence reduction	Gong et al., 2013 [136]
	In vitro LM8, MG-63, Saos2, TC-71 In vivo (mouse)	HMME-PDT	Apoptosis caspase-dependent	Zeng et al., 2013 [148]
	In vitro DLM-8, Saos2, HOS, 143B, U-2 OS In vivo (mouse)	Hiporfin-PDT	Inhibition of proliferation by G2M arrest, ROS increase, Apoptosis and necrosis	Sun et al., 2015 [144]
	In vitro TC-71 In vivo (mouse)	BCDP-18-PDT	Inhibition of proliferation by G2M arrest; apoptosis	Sun et al., 2016 [145]
	In vitro 143B, K7M2L2 In vivo (mouse)	Foscan or Foslip-PDT	Apoptosis Pulmonary metastasis inhibition	Meier et al., 2017 [155]
	In vitro MNNG, MG63 In vivo(mouse)	Magnetic calcium silicate/chitosan porous -PDT	Cytotoxicity Reduction of tumor size	Lu et al. 2018 [159], Yang et al., 2018 [160]
	In vitro MNNG/HOS, U-2OS, MG63, Saos2 In vivo(mouse)	PPZ-PDT	Ros increase, Apoptosis, reduction of cell invasion capacity. Tumor size reduction	Yu et al., 2018 [153]
	In vitro MNNG/HOS, MG63, K7M2 In vivo(mouse)	ZnPc/BSA-PDT	Ros increase, Autophagy, Apoptosis, reduction of cell invasion capacity. Inhibition of tumor growth after surgery	Yu et al., 2019 [154]
In vivo	In vivo (dog)	verteporfin-PDT	Necrosis	Burch et al., 2009 [151]
	In vivo (mouse)	5,15-bis(2-bromo-5-hydroxyphenyl) porphyrin-PDT	Tumor size reduction. Increase of tumor necrosis areas and osteoid matrix volumes	De Miguel et al., 2018 [156]
Clinical trial	10 patients with primary musculoskeletal sarcomas: six with primary malignant soft tissue sarcoma and four with primary malignant bone tumor (two OS)	AO-PDT AO-PDT + irradiation	Results: AO-PDT + irradiation: no recurrence development AO-PDT alone: 1/5 case of recurrence after 21 months Complications: None of the patients clinically showed local or systemic complications caused by AO administration.	Kusuzaki et al., 2005 [161]

Abbreviation: PDT Photodynamic Therapy, AO Acridine Orange, hALA Aminolevulinic acid hexyl ester, mTHPC 5,10,15,20-tetrakis(*meta*-hydroxyphenyl)chlorine, NPe6 N-aspartylchlorin e6, LLLT Low-Level Light Therapy, ALA 5-Aminolevulinic acid, TiO_2_ @xGd NBs Gd-doped TiO_2_ nanobeads, PTX-Ce6@Ker paclitaxel loaded in keratine nanoparticles functionalized with the photosensitizer chlorin-e6, BCDP benzochloroporphyrin derivative, HMME Hematoporphyrin Monomethyl Ether, PPZ Zinc Phthalocyanine, ZnPc/BSA Zinc Phthalocyanine Bovine Serum Albumin conjugated.
